# Digitally Controlled Oscillator with High Timing Resolution and Low Complexity for Clock Generation †

**DOI:** 10.3390/s21041377

**Published:** 2021-02-16

**Authors:** Duo Sheng, Wei-Yen Chen, Hao-Ting Huang, Li Tai

**Affiliations:** Department of Electrical Engineering, Fu Jen Catholic University, New Taipei City 24205, Taiwan; m10907401@mail.ntust.edu.tw (W.-Y.C.); 407216202@mail.fju.edu.tw (H.-T.H.); 407216135@gapp.fju.edu.tw (L.T.)

**Keywords:** digitally controlled oscillator (DCO), clock generator, all digital, low power, low complexity

## Abstract

This paper presents a digitally controlled oscillator (DCO) with a low-complexity circuit structure that combines multiple delay circuits to achieve a high timing resolution and wide output frequency range simultaneously while also significantly reducing the overall power consumption. A 0.18 µm complementary metal–oxide–semiconductor standard process was used for the design, and measurements showed that the chip had a minimum controllable timing resolution of 4.81 ps and power consumption of 142 µW with an output signal of 364 MHz. When compared with other designs using advanced processes, the proposed DCO demonstrated the best power-to-frequency ratio. Therefore, it can output a signal at the required frequency more efficiently in terms of power consumption. Additionally, because the proposed DCO uses digital logic gates only, a cell-based design flow can be implemented. Hence, the proposed DCO is not only easy to implement in different processes but also easy to integrate with other digital circuits.

## 1. Introduction

In digital chips, all circuits rely on clock signals for signal synchronization and coordination to ensure correct timing and functional operation. Therefore, generating high-quality clock signals in the chip to meet the requirements of digital systems is a very important topic. After much research and development, the phase-locked loop (PLL) is currently the most common and important clock generator. PLLs are often used to generate clock signals required by digital systems (e.g., clock multipliers) or in communication systems and data transmission (e.g., frequency synthesizer, clock and data recovery, and clock de-skew). Hence, PLLs are an indispensable and important module in today’s chips [1,2].

**Figure 1:**
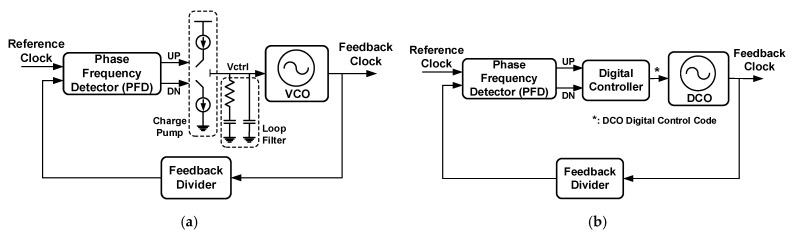
(**a**) Functional block diagram of a general phase-locked loop (PLL); (**b**) Functional block diagram of a general all-digital PLL (ADPLL).

The general PLL design implements the charge pump structure [3,4]. Figure 1a illustrates a functional block diagram of a charge pump PLL, which comprises a phase frequency detector (PFD), charge pump, loop filter, voltage-controlled oscillator (VCO), and feedback divider. The PFD compares the phases of the input reference clock and feedback clock in the feedback divider and determines the period for the charge pump to charge or discharge the capacitor according to the phase difference. Therefore, the charge pump and loop filter determine the control voltage (*V*_ctrl_) of the VCO, and thus, its output frequency. Although the charge pump PLL has good performance, it has encountered several design challenges with the evolution of the semiconductor manufacturing process and reduction of the system operating voltage. For instance, reducing the system operating voltage requires more design efforts to balance the frequency-to-voltage gain and output frequency range in the VCO [5]. Additionally, the capacitors that store the control voltage are implemented with a metal–oxide–semiconductor (MOS) in consideration of the chip cost and area. Furthermore, advanced processes can cause a serious leakage current in the MOS, which can cause the control voltage to generate a ripple phenomenon that in turn generates a jitter in the output clock signal. Thus, several studies have proposed implementing an all-digital PLL (ADPLL) to overcome the challenges faced by traditional PLLs [5,6,7].

The ADPLL is a purely digital circuit that is constructed to realize the PLL. Because all circuits of the ADPLL are controlled by digital signals, the problem of a control voltage ripple caused by a leakage current can be avoided. Additionally, because the ADPLL does not use any passive components, it is very easy to integrate with other digital systems. Such an all-digital circuit can be designed as a soft silicon intellectual property (soft IP), so the design can be easily converted for implementation in different processes, which will significantly reduce the chip development time. Figure 1b illustrates a functional block diagram of a general ADPLL, which comprises a PFD, digital controller, digitally controlled oscillator (DCO), and feedback divider. The ADPLL operates as follows: the PFD compares the phases and frequencies of the input reference clock signal and the feedback clock signal from the feedback divider. It then generates UP and DN signals and sends them to the digital controller. The digital controller adjusts the digital control code for the DCO according to the UP/DN signals and thus controls the DCO output signal frequency. The ADPLL and PLL operate very similar except that the ADPLL circuit is controlled by digital signals instead of a voltage or current. The circuit is implemented with digital logic circuits, and digital control codes are used to determine the frequency of the generated clock signal.

Among the ADPLL modules, the DCO has the greatest effect on the overall performance and thus is the most important circuit [7,8,9,10,11]. Generally, the important performance indices of the ADPLL are the jitter, output frequency range, and power consumption. The jitter represents the frequency stability of the generated clock signal. Because the output clock frequency of the DCO is controlled by the digital control code discretely, changing the digital control code during ADPLL operation changes the output signal frequency slightly. Hence, the jitter of the ADPLL is not only related to the operating environment and locking algorithm of the chip but is also affected by the timing resolution of the DCO. The timing resolution is the relationship between the delay value and the digital control code. When the timing resolution is high, changing the DCO digital control code changes has less of an effect on the output signal frequency, which reduces the jitter. The output clock frequency range of the ADPLL is determined directly from the DCO output frequency range. To expand the applicable range of an ADPLL, a wider output clock frequency range is required, which in turn necessitates a DCO with a wide output clock frequency range. Given that the DCO takes up more than 50% of the overall power consumption of the ADPLL, reducing the power consumption of the DCO is an important design consideration for ADPLLs used in portable or Internet of Things (IoT) devices [2].

Because the essential component of a DCO is the digitally controlled delay element (DCDE), we focus on the design of DCDE in the review of DCO architecture. The main design concept of DCDE is to control the driving current or circuit loading by digital control code to obtain different delay times and generate different output periods. There are four types of DCDE structures that will be reviewed in the next section in detail.

This study focused on developing a DCO with a high timing resolution and wide output clock frequency range realized by standard cells. A low-complexity architecture is proposed to reduce the power consumption and area of the DCO. The rest of this paper is organized as follows. In Section 2, the proposed DCO architecture is briefly explained and compared with recent designs in the literature. In Section 3, the proposed DCO architecture and circuit are described in detail. In Section 4, the chip implementation and measurement results are presented, and the performances of different designs are compared. The paper is concluded in Section 5.

## 2. Review of Digitally Controlled Delay Elements

**Figure 2:**
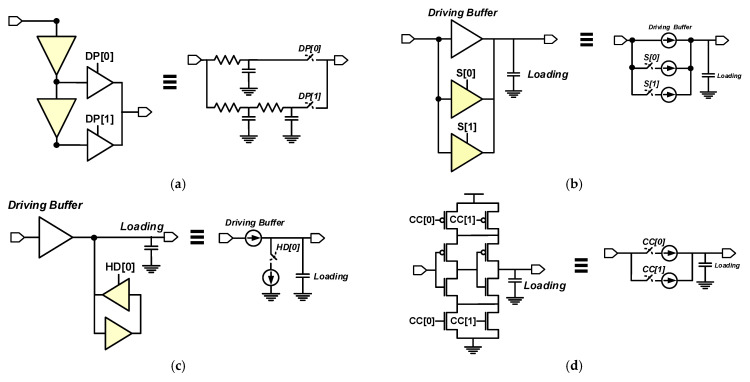
The circuit structure of (**a**) delay-path-type-digitally controlled delay element (DP-DCDE); (**b**) shunt-type (S)-DCDE; (**c**) hysteresis-delay (HD)-DCDE; (**d**) current-controlled (CC)-DCDE.

The essential component of a DCO is the digitally controlled delay element (DCDE). Several studies have proposed different designs for the DCDE. Figure 2 shows four types of DCDE structures: the delay-path-type DCDE (DP-DCDE) [5], shunt-type DCDE (S-DCDE) [11], hysteresis-delay DCDE (HD-DCDE) [8], and current-controlled DCDE (CC-DCDE) [12]. The first three types can be implemented with digital logic gates and completed through a cell-based design flow. The last DCDE is implemented through a full-custom design flow.

The DP-DCDE has a very straightforward design concept: the delay is changed by adjusting the path length of the signal transmission [5]. Delay elements with fixed delays are connected in series to form a delay line, and the output of each delay element is connected to a tri-state gate. Then, the outputs of all tri-state gates are connected together, as shown in Figure 2a. A single tri-state gate is turned on by a specific digital control code at a time, which results in a specific delay path and thus generates a specific delay. The delay range may be widened by increasing the number of cascaded DCDEs, but the delay resolution is a logical gate delay, which is not suitable for general applications.

To increase the delay resolution, the S-DCDE [11] connects several tri-state gates in parallel. The tri-state gates are controlled by digital control codes to obtain different driving capabilities and thus different delay times. Figure 2b shows the circuit structure. The S-DCDE offers a high delay resolution, but increasing the number of turned on tri-state gates reduces the change in driving capability. The S-DCDE eventually becomes saturated, which results in poor linearity between the digital control code and delay.

The HD-DCDE uses digital control codes to control delay hysteresis and generate different delays. Its structure is shown in Figure 2c [5,8]. Although it can realize a delay resolution of less than 100 ps, which is better than that of the DP-DCDE, it is still insufficient for ADPLL applications.

Contrary to the other three designs, the CC-DCDE controls the current analogously to change the delay of the circuit [12]. There are some header and footer switches in the CC-DCDE, and the number of turned-on header and footer switches is controlled by a digital control code, as shown in Figure 2d. As the number of turned-on header and footer switches increase, the supply current of the center inverter increases, leading to reduce the delay of DCDE. Because the header and footer switches are implemented by the transistors directly, the DCDE of [12] has to be implemented in the transistor-level design, and it can not be implemented by the cell-based (logical) flow. Compared with the other three designs, the CC-DCDE has a higher timing resolution, but it also has a higher power dissipation and is difficult to integrate into digital chip designs.

Table 1 briefly compares the performances of the four DCDE designs. None of the designs offer good performance in all aspects. Therefore, the proposed DCO architecture uses different DCDE designs to effectively reduce the circuit complexity and power consumption at the same time.

## 3. Digitally Controlled Oscillator Design

**Figure 3:**
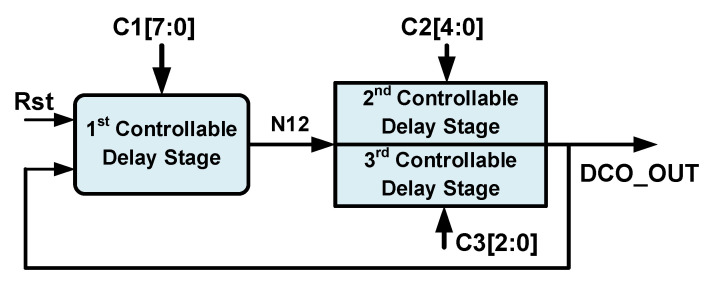
The proposed DCO architecture.

For DCO design, the timing resolution is one of the most important indices for evaluating the performance. However, a high timing resolution is often accompanied by a narrow controllable timing range. Therefore, achieving both a high timing resolution and a wide controllable timing range was the first design challenge in this study. Since using a single DCDE type to achieve both a high timing resolution and a wide controllable timing range at the same time is difficult, the proposed DCO architecture has multiple stages containing different DCDE types. Figure 3 illustrates the proposed DCO architecture, which comprises three different controllable delay stages (CDSs). The delays of the first, second, and third CDSs are controlled by the first, second, and third digital control codes (C1 [7:0], C2 [4:0], and C3 [2:0]), respectively [7]. The first CDS has the highest timing resolution, followed by the second and then third CDSs. Conversely, the third CDS has the widest controllable timing range, followed by the second and then first CDSs. The proposed DCO can obtain both a high timing resolution and a wide controllable timing range through appropriate control because the three CDSs have different timing resolutions and controllable timing ranges. The overall timing resolution of the DCO is that of the third CDS, and the controllable timing range of the DCO can easily be extended from that of the first CDS.

**Figure 4:**
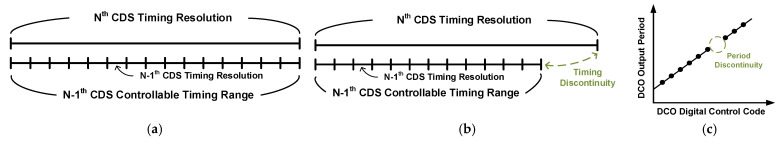
(**a**) Total controllable timing range of a single stage is equal to the timing resolution of the previous controllable delay stage (CDS); (**b**) Total controllable timing range of a single CDS is less than the timing resolution of the previous CDS; (**c**) A large period difference occurs when the control code sweeps across different CDSs.

The main design concept of the multistage DCO is that the total controllable timing range of each stage should be greater than or equal to the timing resolution of the previous stage, as shown in Figure 4a. Thus, the three stages can be effectively combined in a series connection to achieve the design goal. For example, the controllable timing range of the third CDS should be greater than or equal to the timing resolution of the second CDS. Ideally, the same change in the digital control code should produce the same change in the output clock period. However, if the total controllable timing range of a single CDS is less than the timing resolution of the previous CDS, changing some specific digital control codes will cause a greater change in the output clock cycle than other code changes. In such situations, the output clock period controlled by the digital control code will have a serious discontinuity problem, which will increase the output jitter. Figure 4b illustrates a situation where the total controllable timing range of a single CDS is less than the timing resolution of the previous CDS. As shown in Figure 4c, a large period difference occurs when the control code sweeps across different CDSs.

**Figure 5:**
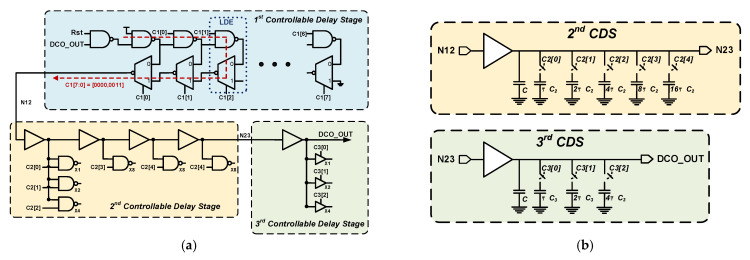
(**a**) The detailed circuit of the proposed multistage digitally controlled oscillator (DCO); (**b**) The equivalent circuits of the second and third CDSs.

Figure 5a shows the detailed circuit of the proposed multistage DCO. The first CDS comprises a NAND gate and eight ladder delay elements (LDEs), each of which contains an inverting multiplexer and NAND gate. There are eight different propagation paths from the input (DCO_OUT) to the output (N12) in the first CDS. The first digital control code (C1 [7:0]) can select one of eight different propagation paths to generate the required delay. Since the first CDS has a ladder-like structure, the overall controllable timing range can be increased by increasing the number of connected LDEs. Compared with the conventional DP-DCDE, the LDE has two advantages. First, the output capacitance increases with the number of delay elements because every delay element output connects together in the conventional DP-DCDE. Hence, if the number of delay elements is increased to extend the controllable timing range, then the instinct delay increases, which decreases the maximum output frequency. By contrast, the instinct delay of the LDE does not increase as the number of delay elements increases, so the maximum output frequency does not decrease when the controllable timing range is extended. Second, the first digital control code not only determines the signal propagation path but also blocks useless signal transmission to reduce unnecessary power consumption.

The design concept of the second and third CDSs is to control the gate capacitance of the logic gate to generate different delays. The gate capacitance is changed very slightly by the digital control code, so the second and third CDSs can have very high timing resolutions. In the second CDS, one input of the two-input NAND gate connects to the delay line, and the other input connects with the digital control code. As the logic level of the digital control code changes, the gate capacitance of the two-input NAND gate changes. This changes the capacitance loading of the delay line in the second CDS. The circuit structure of the third CDS is similar to that of the second CDS; it comprises several tri-state gates where the gate capacitance changes with the logic level of the input. Figure 5b illustrates the equivalent circuits of the second and third CDSs, and the different control signal states obtain different gate capacitances, which generate different delay times. The second and third CDSs have 32 and eight different delays, respectively, that are controlled by the second digital control code (C2 [4:0]) and third digital control code (C3 [2:0]), respectively. Because the third CDS has a very high timing resolution, using only the first and third CDSs would cause the third CDS to require a large number of tri-state gates to ensure that its controllable timing range is greater than the timing resolution of the first CDS. Therefore, to greatly reduce the number of tri-state gates in the third CDS, the second CDS is added with a timing resolution lower than that in the third CDS. Thus, the proposed multistage DCO has not only a high timing resolution but also low circuit complexity and power consumption.

Table 2 lists the HSPICE simulation results for the timing resolution and range of each CDS. The proposed DCO was simulated with a 0.18 µm complementary MOS (CMOS) model under three different process–voltage–temperature (PVT) conditions: best-case (FF, 1.98 V, −40 °C), typical-case (TT, 1.8 V, 25 °C), and worst-case (SS, 1.62 V, 125 °C). According to the simulation results, each stage had a greater total controllable timing range than the timing resolution of the previous stage under all PVT conditions. Thus, the three stages can be effectively connected in series to achieve the design goal.

## 4. Measurement Results and Discussion

**Figure 6:**
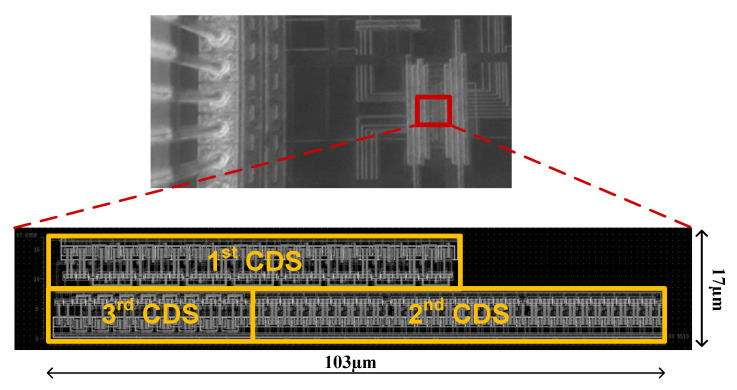
Microphotograph and layout of DCO chip.

**Figure 7:**
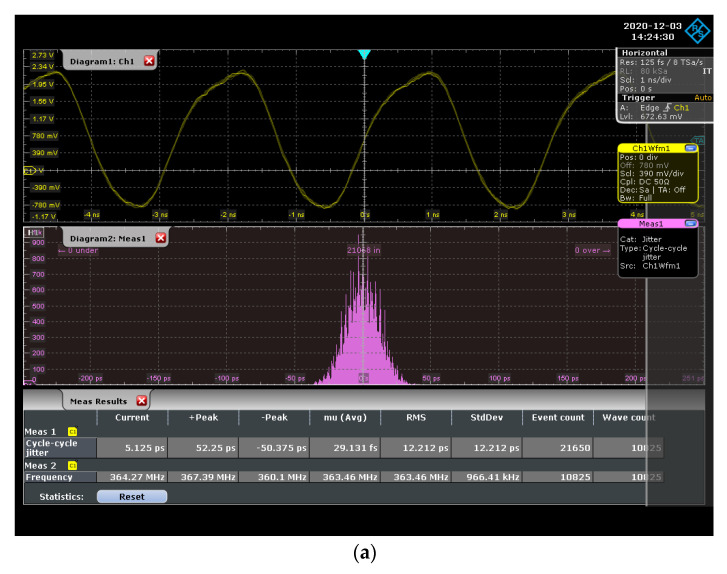

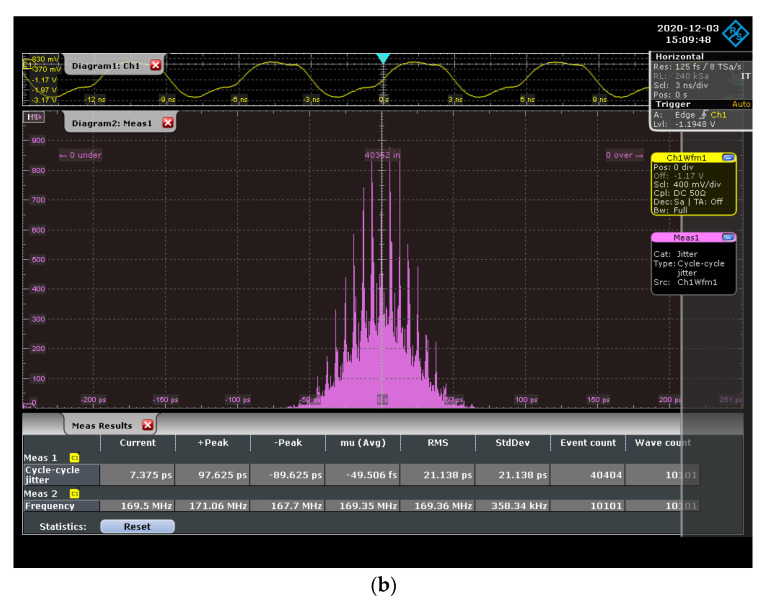
Chip measurement results of (**a**) at 364 MHz; (**b**) at 170 MHz.

The proposed DCO was designed through the cell-based design flow and implemented according to the TSMC 0.18 µm 1P6M CMOS standard process. Figure 6 shows a microphotograph and layout of the chip, which had an area of 17 µm × 103 µm. The DCO chip was measured with an R&S ROT1044 oscilloscope at a supply voltage of 1.8 V and temperature of 25 °C. We use an oscilloscope to measure the different signal frequencies output when inputting different digital control codes, and then obtain the average resolution of the DCO chip. Figure 7 mainly shows two DCO chip measurement results, one is the output frequency range of the proposed DCO, and the other is the jitter at the highest and lowest output frequency, respectively. The measured output frequency range and period resolutions were 170–364 MHz and 4.81 ps, respectively. Figure 7 shows that the root-mean-square jitter was 12.2 and 21.3 ps at 364 and 170 MHz, respectively. The power of the DCO chip can be obtained from the measured current magnitude by an ammeter. According to the maximum power consumption and output frequency of the DCO chip, the overall power index of the proposed DCO can be calculated (0.142 mW/364 MHz = 0.39 μW/MHz).

Table 3 compares the performances of the proposed DCO design and state-of-the-art DCOs. Because of the low circuit complexity of the proposed DCO, although the process was not as advanced as that used in the other designs, it had the smallest chip area. Additionally, the power index results clearly demonstrate that the proposed DCO had the best power-to-frequency ratio, which implies that it reduces power consumption more effectively for a given output frequency. Because all circuits of the proposed DCO can be implemented using logic gates, it can be designed with a cell-based design flow. This not also can reduce the chip design time significantly but also allows the circuit to be easily migrated to a different process.

## 5. Conclusions

This paper presents a DCO with high timing resolution and low complexity for clock generation. The proposed multistage DCO comprises three CDSs to achieve a high timing resolution with low power consumption and circuit complexity. Measurements showed that the proposed DCO reduced the power consumption to 0.142 mW at 364 MHz with a period resolution of 4.81 ps. The proposed DCO implements a cell-based design flow that is easy to integrate with digital systems, and it can be migrated to different technologies. The proposed DCO can be used to realize an area- and power-efficient clock generator for advanced system applications.

## Figures and Tables

**Table 1:** Comparisons of different digitally controlled delay element (DCDE) structures.

	DP-DCDE [5]	S-DCDE [11]	HD-DCDE [8]	CC-DCDE [12]
Design style	Cell-based	Cell-based	Cell-based	Full-custom
Timing resolution	Low	Medium	Medium	High
Delay Linearity	Good	Poor	Good	Poor
Operation range	Wide	Narrow	Medium	Narrow
Power consumption	Medium	Medium	Medium	High
Portability	Yes	Yes	Yes	No

**Table 2:** The timing resolution and controllable range of each CDS.

	First CDS	Second CDS	Third CDS
Range (ps)	2647.9	435.5	35.2
Resolution (ps)	382.1	14.5	4.4

**Table 3:** Performance Comparisons.

Performance Indices	Proposed	RSI’20 [11]	TCAS2’11 [6]	TVLSI’17 [10] ^1^	TCAS2’07 [5]	JSSC’05 [12]
Process	0.18 μm CMOS	0.18 μm CMOS	65 nm CMOS	65 nm CMOS	90 nm CMOS	0.18 μm CMOS
Supply Voltage (V)	1.8	1.8	1	1.2	1	1.8
Design Methodology	All-Digital (Cell-Based)	All-Digital (Cell-Based)	All-Digital (Cell-Based)	Semi-Digital	All-Digital (Cell-Based)	Full-Custom
Operation Range (MHz)	170~364	169~522	47.8~538.7	600~3100	191~952	413~485
Resolution	4.81 ps	2.2 ps	17.4 ps	4.45~5.06 (MHz/LSB)	1.47 ps	2 ps
Power Consumption (mW)	0.142 @364 MHz	1.8 @519 MHz	0.205 @481.6 MHz	2.4 @1.87 GHz	0.14 @200 MHz	0.17~0.34 ^2^
Power Index (μW/MHz)	0.39	3.47	0.43	1.28	0.7	NA
Area (μm × μm)	103 × 17	55 × 55	0.01 mm^2^	NA	NA	100 × 50
Portability	Yes	Yes	Yes	No	Yes	No

^1^ Simulation only, ^2^ Static power only.

## Data Availability

Not applicable.
